# Unmasking Orbital Myopathy: When Enlarged Extraocular Muscles Indicate More Than Thyroid Eye Disease: Report of Two Cases

**DOI:** 10.1155/crop/7712244

**Published:** 2026-09-11

**Authors:** Daisuke Shinohara, Muhammad Abumanhal, Yasuhiro Takahashi

**Affiliations:** ^1^ Department of Oculoplastic, Orbital and Lacrimal Surgery, Aichi Medical University Hospital, Nagakute, Japan, aichi-med-u.ac.jp; ^2^ Department of Ophthalmology, Tel Aviv Sourasky University Medical Center (Ichilov), Tel Aviv, Israel

**Keywords:** breast cancer metastasis, immunoglobulin G4-related ophthalmic disease, orbital myopathy, thyroid eye disease

## Abstract

**Introduction:**

Orbital myopathy is a condition involving the extraocular muscles, and establishment of a differential diagnosis is crucial to determine its etiology. However, the broad spectrum of the etiologies and the overlap in their clinical and radiological findings often make the diagnosis challenging. We report two cases of orbital myopathy that clinically mimicked thyroid eye disease (TED).

**Case Presentation:**

Both patients underwent unsuccessful medical ± surgical treatments, based on the presumed diagnosis of TED at other institutions. One patient presented with enlargement of multiple extraocular muscles, causing proptosis and extraocular motility restriction, whereas the other showed inferior rectus muscle enlargement and subsequent severe restrictive hypotropia. Incisional biopsy was performed at our institution in both cases: Lacrimal gland biopsy confirmed a histopathological diagnosis of immunoglobulin G4‐related ophthalmic disease in one patient, whereas extraocular muscle biopsy revealed metastasis from breast carcinoma in the other.

**Discussion:**

These cases underscore the importance of comprehensive diagnostic approach, including consideration of alternative etiologies and tissue biopsy, to ensure accurate diagnosis of orbital myopathy.

## 1. Introduction

Orbital myopathy is a clinical condition characterized by involvement of the extraocular muscles (EOMs) rather than a diagnosis itself. Although thyroid eye disease (TED) is the most common cause, a wide spectrum of other conditions, including orbital inflammatory diseases, vascular diseases, infections, neoplasms, and other entities can present with orbital myopathy [1–4]. Therefore, identifying the underlying etiology requires a systematic differential diagnostic approach, based on clinical findings, laboratory tests, and orbital imaging, rather than assuming TED as the default diagnosis. Because many of these conditions share overlapping clinical and radiological features, establishing the correct diagnosis can be challenging and may occasionally require additional systemic evaluation and tissue biopsy.

Here, we report two patients who had been initially diagnosed and treated as TED at other clinics but were later diagnosed as immunoglobulin G4‐related ophthalmic disease (IgG4‐ROD) and metastasis from breast cancer.

## 2. Case Presentation

The manuscript was checked against the CARE guidelines.

### 2.1. Case 1

A 71‐year‐old man with pulmonary lesions suspected to represent sarcoidosis and no family history of thyroid disease presented with a 1‐year history of bilateral eyelid swelling and conjunctival injection. Magnetic resonance images (MRI) showed tendon‐sparing bilateral enlargement of the inferior and medial rectus muscles, along with enlargement of the right lateral rectus and inferior oblique muscles. The EOM enlargement was accompanied by mild high intensity on T2‐weighted images. Based on these imaging findings, the patient was diagnosed with active TED by an ophthalmologist and a radiologist and was treated with weekly 500 mg of intravenous methylprednisolone for 10 weeks. Although his symptoms transiently improved during the treatment, they recurred immediately after its completion. Then, the patient underwent subtenon triamcinolone injection followed by additional course of weekly intravenous methylprednisolone (500 mg) for 6 weeks, with a similar clinical course.

**Figure 1:**
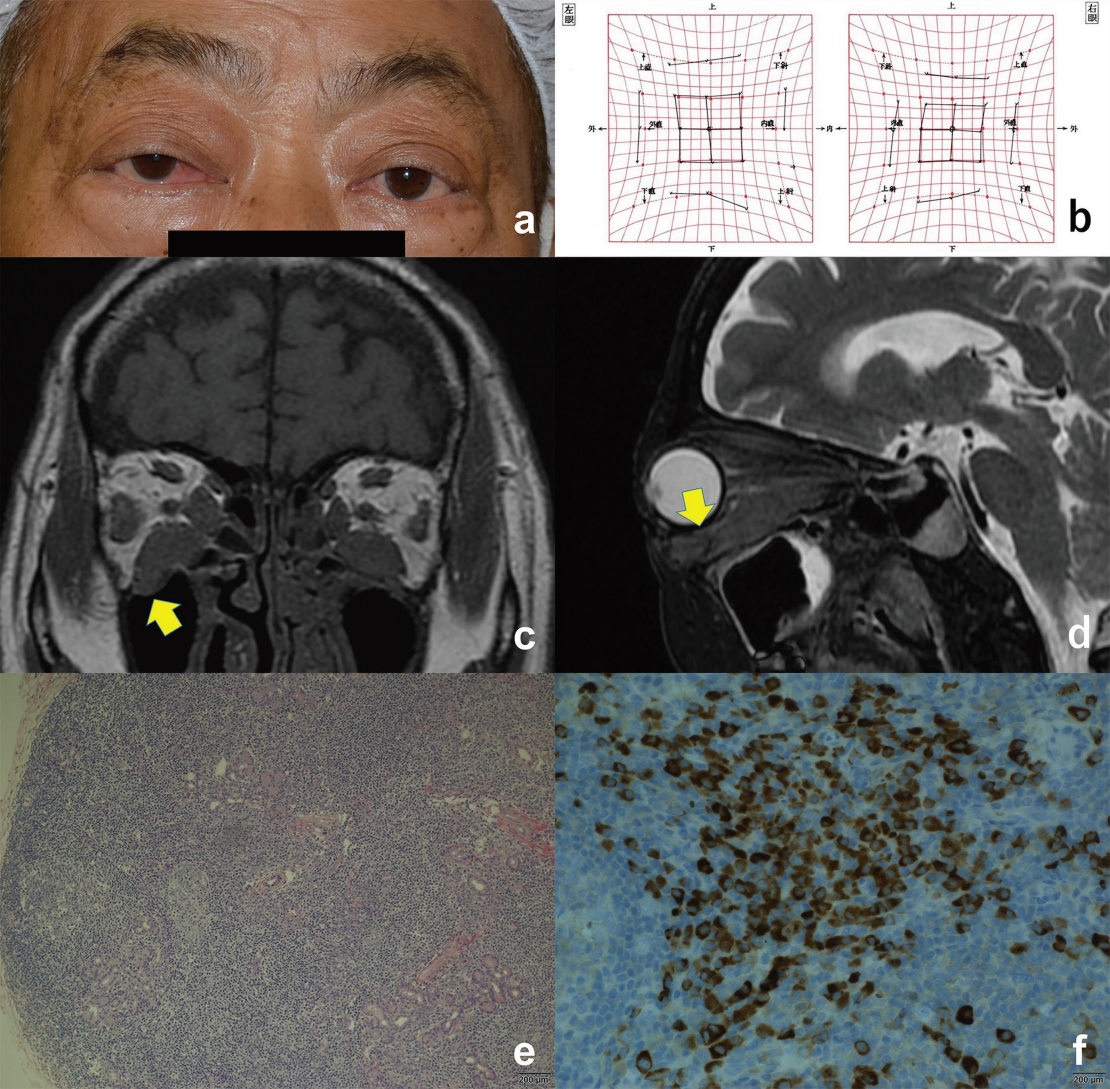
Case 1. (a) Facial photograph showing bilateral mild‐to‐moderate proptosis and ptosis. (b) Hess chart showing mild restriction of elevation, adduction, and abduction in the right eye. (c, d) Magnetic resonance imaging (MRI). (c) A coronal T1‐weighted magnetic resonance image showing enlargement of the inferior and medial rectus muscles bilaterally and the right lateral rectus muscle. An enlarged right infraorbital nerve (arrow) is contiguous with the enlarged inferior rectus muscle. (d) A sagittal fat‐suppressed image showing enlargement of the inferior rectus and inferior oblique muscles. The inferior rectus muscle tendon is not involved (arrow). (e, f) Histopathological examination. (e) Marked lymphocytic infiltration (hematoxylin and eosin staining; magnification, × 40). (f) Immunohistochemistry showing marked immunoglobulin G4‐positive cell infiltration (magnification, × 400).

At the initial examination at our institution, the best‐corrected visual acuity was 0.9 in both eyes. Slit lamp examination revealed superior limbic keratoconjunctivitis in both eyes. Margin reflex distance‐1 (MRD‐1) was 0.5 mm on the right side and 2.0 mm on the left side (Figure 1a). MRD‐2 was 6.5 mm on the right side and 5.5 mm on the left side. Hertel exophthalmometric value was 18.0 mm on the right side and 16.0 mm on the left side (base, 101 mm). Hess chart showed mild restriction of elevation, adduction, and abduction in the right eye (Figure 1b). MRI showed enlargement of the inferior rectus, medial rectus and inferior oblique muscles on both sides, right lateral rectus muscle, and left lacrimal gland (Figures 1c,d). An enlarged right infraorbital nerve was contiguous with the enlarged inferior rectus muscle. Blood test showed that thyroid hormones were within normal range, and thyroid autoimmune antibodies were negative.

Based on those findings, IgG4‐ROD, rather than TED was suspected. Repeated blood testing demonstrated marked elevations of serum IgG4 (925 mg/dL; normal range, < 135.0 mg/dL) and soluble interleukin‐2 receptor (929 U/mL; normal range, 122–496 U/mL). Systemic computed tomographic (CT) images showed bilateral hilar and mediastinal lymphadenopathy. An incisional biopsy of the left lacrimal gland was performed. Histopathology examination demonstrated dense lymphocytic infiltration. Immunohistochemistry revealed IgG4‐positive cell staining with more than 50 IgG4‐positive plasma cells per high‐power field and IgG4‐positive/IgG‐positive cell ratio of 77% (Figures 1e,f), fulfilling the diagnostic criteria for IgG4‐ROD [5]. Oral prednisolone at dose of 40 mg daily was initiated, and ptosis, proptosis, and extraocular motility improved rapidly. At 2.5 years follow‐up, the patient continued maintenance therapy with prednisolone of 8 mg daily, with sustained improvement in the periocular findings. Serum IgG4 level remained stable within the range of 160–180 mg/dL.

### 2.2. Case 2

A 53‐year‐old woman with no personal or family history of thyroid disease presented with a 2‐year history of vertical diplopia. She was initially diagnosed with TED by an ophthalmologist, based on enlargement of the left inferior rectus and bilateral medial rectus muscles and weak positivity for thyroid autoimmune antibodies. After steroid pulse therapy, she underwent left inferior rectus muscle recession. Since her diplopia did not improve at all, additional recession of the left inferior and medial rectus muscles was performed 1 year later. Despite these interventions, she continued to have severe hypotropia with consequent diplopia. The patient had undergone surgery for right breast carcinoma 8 years ago and had a plan to receive chemotherapy for a metastatic right femur lesion.

**Figure 2:**
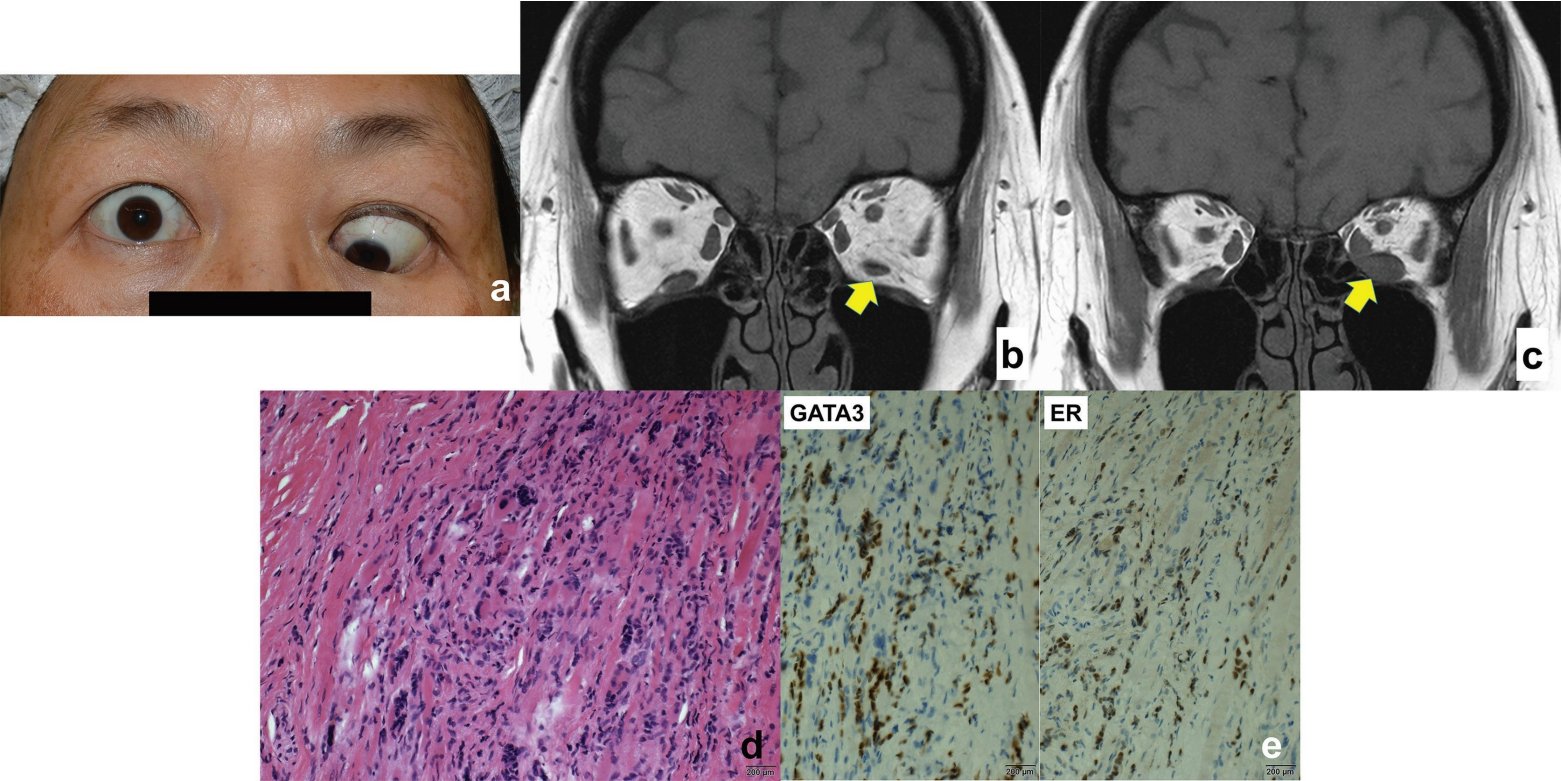
Case 2. (a) A patient face photo showing severe hypotropia in the left eye. (b, c) Coronal T1‐weighted magnetic resonance images showing no enlargement of the inferior rectus muscle in (b) the anterior segment but marked enlargement in (c) the posterior segment (arrows). (d) Histopathological examination showing tumor cell proliferation and infiltration in a restiform rearrangement between muscle fibers (hematoxylin and eosin staining; magnification, × 200). (e) Immunohistochemical staining shows that tumor cells are positive for GATA‐binding protein 3 and estrogen receptor (ER) (magnification, × 200).

At her initial examination at our institution, the best‐corrected visual acuity was 0.9 in the right eye and 0.4 in the left eye. Hertel exophthalmometric value was 14.0 mm on the right side and 15.0 mm on the left side (base, 95 mm). Her left eye was severely deviated inferiorly (Figure 2a), and Hess chart was not feasible due to the severe hypotropia. Previous MRI from another clinic (sagittal sections were not taken) demonstrated enlargement of the left inferior and bilateral medial rectus muscles, with the inferior rectus muscle enlargement most pronounced in the posterior segment (Figures 2b,c). Blood test revealed normal thyroid hormone levels and negativity for thyroid autoimmune antibodies.

The focal enlargement of the inferior rectus muscle raised suspicion for breast cancer metastasis rather than TED. Repeat blood tests revealed markedly elevated tumor markers, including carcinoembryonic antigen (CEA; 319.1 ng/mL: normal range, < 5.0 ng/mL) and cancer antigen 15‐3 (CA 15‐3; 63.5 U/mL; normal, < 25.0 U/mL). An incisional biopsy of the left inferior rectus muscle was performed. Histopathological analysis showed proliferation of AE1/AE3 and CAM5.2‐positive cells between skeletal muscle fibers (Figure 2d). The atypical cells were positive for GATA‐binding protein 3 and estrogen receptor, confirming metastatic breast carcinoma. The patient is currently undergoing chemotherapy as planned.

## 3. Discussion

We report two patients with enlarged EOMs who were initially misdiagnosed and treated as TED. In TED, EOM enlargement typically exhibits a fusiform shape, and the affected enlarged contracted muscles often cause moderate‐to‐severe restriction of ocular motitlity [6, 7]. Additionally, positivity of thyroid autoantibodies is generally supportive of the diagnosis of TED [8].

Although Case 1 presented with inflammatory signs including eyelid swelling and conjunctival injection, and radiologic enlargement of the EOMs with mild high intensity lesions, resembling active TED, thyroid autoantibodies were negative. In addition, restriction of extraocular motility was mild despite severely enlarged EOMs, consistent with the relative preservation of orbital soft tissue function typically observed in IgG4‐ROD [9, 10]. TED has active and inactive (chronic) phases, and anti‐inflammatory treatment, such as intravenous methylprednisolone and teprotumumab, is generally indicated for active TED [11]. However, the rapid recurrence of symptoms following intravenous methylprednisolone therapy further argued against active TED [12]. Long‐term oral prednisolone administration is generally necessary for the management of IgG4‐ROD [13]. Moreover, the presence of an enlarged infraorbital nerve is highly suggestive of IgG4‐ROD [14]. However, in this case, the right inferior rectus muscle was also enlarged and appeared to be integrated with the infraorbital nerve, which may explain why the enlargement of the infraorbital nerve was overlooked at the previous clinic.

In Case 2, the weak positivity for thyroid autoantibodies and the presence of restrictive hypotropia initially suggested TED. On the contrary, this restrictive hypotropia was extremely severe and did not improve after two strabismus surgeries. Although there had been no comparative data regarding the degree of extraocular motility restriction between TED and metastatic tumors in the EOMs, extraocular motility impairment tends to be severe in cases with metastatic tumors [15, 16]. Additionally, MRI demonstrated that the left inferior rectus muscle was predominantly enlarged in the posterior segment forming a nodular lesion, which is more common in metastatic orbital tumors [3, 4].

Clinical evaluation of both cases revealed several features that challenge the initial diagnosis of TED and should prompt ophthalmologists to reconsider alternative etiologies. Key clues include ptosis rather than the typical lid retraction seen in TED, involvement of other systemic lesions, rapid recurrence of symptoms after completion of steroid therapy, and localized or segmental enlargement of EOMs rather than the diffuse fusiform pattern typical of TED. Additional atypical findings, such as infraorbital nerve involvement and unusually severe or progressive extraocular motility restriction unresponsive to standard interventions, further deviate from the expected clinical course of TED. These atypical features should raise suspicion for other orbital pathologies, warrant repeat laboratory testing, and consideration of tissue biopsy to establish a definitive diagnosis.

In conclusion, the present cases underscore the critical necessity for a comprehensive approach for diagnosis of orbital myopathy, especially for differential diagnosis between TED and other entities.

## Author Contributions

Conceptualization, Y.T.; methodology, Y.T.; data curation, D.S. and Y.T.; validation, Y.T.; formal analysis, Y.T.; investigation, Y.T.; writing—original draft preparation, D.S.; writing—review and editing, M.A. and Y.T.; supervision, Y.T.; project administration, Y.T. No one contributed to the work who did not meet our authorship criteria.

## Funding

No funding was received for this manuscript.

## Disclosure

All authors have read and approved the final version of the manuscript. The corresponding author had full access to all of the data in this study and takes complete responsibility for the integrity of the data and the accuracy of the data analysis. The corresponding author affirms that this manuscript is an honest, accurate, and transparent account of the study being reported; that no important aspects of the study have been omitted; and that any discrepancies from the study as planned (and, if relevant, registered) have been explained. All authors declare that the research was conducted in the absence of any commercial or financial relationships that could be construed as a potential conflict of interest.

## Consent

Written informed consent was obtained from the patients for publication of their face photographs and clinical course.

## Conflicts of Interest

Y.T. received speaker and consultant honoraria from Amgen Inc., Chugai Pharmaceutical Co. Ltd., Santen Pharmaceutical Co. Ltd., and Ono Pharmaceutical Co. Ltd. The disclosed relationships are unrelated to the present work.

## Data Availability

The data that support the findings of this study are available on request from the corresponding author. The data are not publicly available due to privacy or ethical restrictions.
